# Connectivity in MEG resting-state networks increases after resective surgery for low-grade glioma and correlates with improved cognitive performance^☆^

**DOI:** 10.1016/j.nicl.2012.10.007

**Published:** 2012-11-02

**Authors:** E. van Dellen, P.C. de Witt Hamer, L. Douw, M. Klein, J.J. Heimans, C.J. Stam, J.C. Reijneveld, A. Hillebrand

**Affiliations:** a Department of Neurology, VU University Medical Center, Amsterdam, The Netherlands; b Neurosurgical Center Amsterdam, VU University Medical Center, Amsterdam, The Netherlands; c Athinoula A. Martinos Center for Biomedical Imaging, Massachusetts General Hospital, Boston, MA, USA; d Harvard Medical School, Boston, MA, USA; e Department of Clinical Neurophysiology and MEG Center, VU University Medical Center, Amsterdam, The Netherlands; f Department of Medical Psychology, VU University Medical Center, Amsterdam, The Netherlands

**Keywords:** Glioma, Resective surgery, Cognition, Magnetoencephalography, Resting-state networks, Functional connectivity

## Abstract

**Purpose:**

Low-grade glioma (LGG) patients often have cognitive deficits. Several disease- and treatment related factors affect cognitive processing. Cognitive outcome of resective surgery is unpredictable, both for improvement and deterioration, especially for complex domains such as attention and executive functioning. MEG analysis of resting-state networks (RSNs) is a good candidate for presurgical prediction of cognitive outcome. In this study, we explore the relation between alterations in connectivity of RSNs and changes in cognitive processing after resective surgery, as a stepping stone to ultimately predict postsurgical cognitive outcome.

**Methods:**

Ten patients with LGG were included, who had no adjuvant therapy. MEG recording and neuropsychological assessment were obtained before and after resective surgery. MEG data were recorded during a no-task eyes-closed condition, and projected to the anatomical space of the AAL atlas. Alterations in functional connectivity, as characterized by the phase lag index (PLI), within the default mode network (DMN), executive control network (ECN), and left- and right-sided frontoparietal networks (FPN) were compared to cognitive changes.

**Results:**

Lower alpha band DMN connectivity was increased after surgery, and this increase was related to improved verbal memory functioning. Similarly, right FPN connectivity was increased after resection in the upper alpha band, which correlated with improved attention, working memory and executive functioning.

**Discussion:**

Increased alpha band RSN functional connectivity in MEG recordings correlates with improved cognitive outcome after resective surgery. The mechanisms resulting in functional connectivity alterations after resection remain to be elucidated. Importantly, our findings indicate that connectivity of MEG RSNs may be used for presurgical prediction of cognitive outcome in future studies.

## Highlights

► MEG RSN functional connectivity was estimated pre- and post-surgery in LGG patients. ► Alpha band DMN and right FPN functional connectivity was increased after surgery. ► These RSN connectivity increases correlated with improved cognitive functioning. ► Connectivity analysis of MEG RSNs may be used for presurgical planning.

## 1 Introduction

Low-grade glioma (LGG) patients often have cognitive deficits that limit their quality of life. Cognitive performance in these patients is affected by several factors, related to both the disease itself and to its treatment (Klein et al., 2012). The preservation and possibly restoration of cognitive performance are particularly important for this patient population with a relatively long life expectancy. Resective surgery may result in cognitive improvement or deterioration, as the resection removes infiltrative tumor tissue that disturbs the surrounding normal brain tissue, and at the same time disrupts connections to surrounding normal brain that can be critically functional (Klein et al., 2012). Especially the surgical outcome of complex cognitive processes such as attention, executive functioning and memory processing is unpredictable. Improved presurgical prediction of cognitive outcome would significantly contribute to patient counseling and surgical management decisions.

Mapping of functional connectivity patterns may be worthwhile for this purpose. An optimal organization of functional brain networks is required for proper cognitive processing (Bullmore and Sporns, 2012; Stam and van Straaten, 2012). These networks can be analyzed by characterizing functional connectivity between brain areas, using magnetoencephalography (MEG), electroencephalography (EEG) and fMRI recordings (Stam and van Straaten, 2012). In glioma patients, like in most patients with neurological and psychiatric diseases, the organization of functional networks is globally disturbed (Bullmore and Sporns, 2012; Heimans and Reijneveld, 2012; Stam and van Straaten, 2012). We have shown that MEG functional connectivity patterns change after resective brain surgery (Douw et al., 2008). Moreover, other studies have shown that preoperative functional connectivity may be of value to guide surgical management decisions in these patients. An MEG study showed that in healthy subjects, especially alpha band connectivity is high in functionally critical brain areas such as somatosensory and language areas, and that this connectivity is altered in glioma patients (Guggisberg et al., 2008). Two consecutive studies showed that areas with decreased alpha band connectivity could be resected without neurological deficits (Martino et al., 2011; Tarapore et al., 2012). Connectivity analysis of large-scale brain networks therefore seems promising as a tool to predict surgical outcome.

Increased MEG connectivity in the delta, theta and gamma frequency bands has been associated with poorer cognitive performance in brain tumor patients (Bosma et al., 2008). Similarly, glioma patients with a more locally clustered and less integrated network organization (of MEG recordings filtered in the delta and lower alpha frequency bands) perform worse during neuropsychological assessments (Bosma et al., 2009). However, these studies describe global alterations in network organization rather than communication between anatomically defined regions. The current study pertains to specific subnetworks, and not the global network. In healthy subjects, several so-called resting-state networks (RSNs) have been linked in fMRI studies to performance in cognitive domains such as attention, memory processing and motor functioning (Brookes et al., 2011; Damoiseaux et al., 2006; Hipp et al., 2012; Rosazza and Minati, 2011). The default mode network (DMN), which is deactivated during tasks, is the most consistently described RSN, and its resting-state activity has been related to performance in several cognitive domains including attention and working memory (Broyd et al., 2009). Also, a left-sided and a right-sided frontoparietal network (FPN) are thought to be crucial for attention, language and memory processing, while a frontal–cingulate network has been described as the executive control network (ECN) (Cole and Schneider, 2007; Rosazza and Minati, 2011). Correlations between decreased RSN activity and cognitive deficits have been described in several neurological disease states such as Alzheimer's disease, stroke, and epilepsy (Broyd et al., 2009; Nomura et al., 2010). Our study provides novel longitudinal information of both alterations in MEG RSN connectivity and cognitive changes in a population which is not exposed to disease or therapy-related factors associated with these changes, other than resective surgery.

In this study, we aim to determine alterations in RSN connectivity after resective surgery in LGG patients, and relate this to changes in cognition. For this purpose, we study a relatively small but homogenous group of LGG patients that underwent resection without any other interventions or medication changes. We focus on the DMN, FPN and ECN, since these RSNs are involved in complex cognitive domains, which are especially relevant for this patient population. We apply a recently developed beamformer method in combination with the phase lag index (PLI), a phase synchronization measure that is relatively insensitive to common sources (Hillebrand et al., 2012; Stam et al., 2007), to MEG recordings. Based on studies in patients with other neurological pathology, we hypothesize that higher RSN network connectivity after tumor resection in LGG patients is related to better cognitive performance outcome (Broyd et al., 2009; Nomura et al., 2010; Sharp et al., 2011).

## 2 Methods

### 2.1 Patients

Consecutive patients were referred for MEG recordings by the Neurosurgical Center Amsterdam between April 2010 and April 2011. Data were collected as part of the LESION study, which is a prospective longitudinal observational study of patients eligible for tumor surgery. Inclusion criteria were (1) adult (≥ 18 years) patients who (2) had resective surgery for a histopathologically confirmed LGG (WHO grade II) and (3) had no other oncological therapy, and (4) had given written informed consent. MEG recording and neuropsychological assessment were obtained at 2 time points: prior to neurosurgical intervention (T1), and within six months after surgery (T2).

### 2.2 Ethics statement

Ethical approval was granted by the VU University Medical Ethics Committee. All patients had given written informed consent before participating. All clinical investigations were conducted according to the Declaration of Helsinki.

### 2.3 Neuropsychological screening

Based on previous studies in LGG patients, six cognitive domains were defined (Table 1), and each subtest of the neuropsychological assessment was used as a measure for functioning in one or more cognitive domains (Douw et al., 2009a; Klein et al., 2003). A performance z-score for each domain was calculated by comparing each person's score with the mean and standard deviation (SD) of all patients' test scores at T1. The same mean and SD value were used to calculate z-scores at both time points. A delta-score was calculated for each patient by subtracting the z-scores for both time points in order to quantify change in cognitive performance.

### 2.4 Magnetoencephalography (MEG)

MEG recordings were made in a magnetically shielded room (VacuumSchmelze GmbH, Hanua, Germany) using a 306-channel whole-head neuromagnetometer (Elekta Neuromag Oy, Helsinki, Finland). Five minutes of MEG data were recorded during a resting-state eyes-closed condition with a sample frequency of 1250 Hz. An anti-aliasing filter and high-pass filter of 410 Hz and 0.1 Hz were applied, respectively. Other artifacts were removed from the data with an offline spatial filter, namely the temporal extension of Signal Space Separation (tSSS) (Taulu and Hari, 2009; Taulu and Simola, 2006) in MaxFilter software (Elekta Neuromag Oy, version 2.2.10). A sliding window of 10 s was used. Channels that were malfunctioning during the recording, for example due to excessive noise, were automatically discarded before estimation of the SSS coefficients. Additionally, malfunctioning channels were identified by visual inspection of the data, and excluded before applying tSSS. The number of excluded channels varied between one and eight. The tSSS filter was then used to remove noise signals that SSS failed to discard, typically from noise sources near the head, using a subspace correlation limit of 0.9.

The head position relative to the MEG sensors was recorded continuously using the signals from four head-localization coils. The head-localization coil positions were digitized, as well as the outline of the participants scalp (~ 500 points), using a 3D digitizer (3Space FastTrack, Polhemus, Colchester, VT, USA). This scalp surface was used for co-registration with the patient's anatomical MRI.

### 2.5 Anatomical MRI

Structural Magnetic Resonance Images (MRI) were made for co-registration with a sagittal slice distance of 0.5–1.5 mm. Co-registration of these T1-weighted MRIs with the MEG data was achieved using surface matching software developed by one of the authors (AH), resulting in an estimated co-registration accuracy of approximately 4 mm (Whalen et al., 2008). A single best fitting sphere was fitted to the outline of the scalp as obtained from the co-registered MRI, which was used as a volume conductor model for the beamformer analysis described below. The co-registered MRI was then spatially normalized to a template MRI using the SEG-toolbox in SPM8. The new segmentation toolbox (Weiskopf et al., 2011) is an extension of the unified segmentation algorithm (Ashburner and Friston, 2005), which incorporates additional tissue priors for improved matching of the subject's MRI to the template. The AAL atlas was used to label the voxels in a subject's normalized co-registered MRI (Tzourio-Mazoyer et al., 2002). Subcortical structures were removed, and the voxels in the remaining 78 cortical regions of interest (ROIs) were used for further analyses (Gong et al., 2009), after inverse transformation to the patient's co-registered MRI.

### 2.6 Time-series estimation for regions-of-interest

We used the beamformer approach as described by Hillebrand et al. (2012). In summary, neuronal activity in the labeled voxels in the ROIs was reconstructed using a scalar beamformer implementation (Elekta Neuromag Oy, beamformer, version 2.1.27) similar to Synthetic Aperture Magnetometry (Robinson and Vrba, 1999). This beamformer sequentially reconstructs the activity for each voxel in a predefined grid covering the entire brain (spacing 2 mm) by selectively weighting the contribution from each MEG sensor to a voxel's time-series. Each ROI contains many voxels and the number of voxels will be different for each ROI. In order to represent a ROI by a single time-series, we selected, for each ROI and frequency band separately, the voxel with maximum pseudo-Z value in that frequency band (Robinson and Vrba, 1999). For the computation of the pseudo-Z values we estimated the data covariance for, on average, 325 s (range: 144–694 s) of data, and used a unity matrix for the noise covariance. The broad-band (0.5–48 Hz) time-series for these selected voxels were used for further analysis. This resulted in 78 time-series for each frequency band. Six frequency bands were analyzed: delta (0.5–4 Hz), theta (4–8 Hz), lower alpha (8–10 Hz), upper alpha (10–13 Hz), beta (13–30 Hz), and lower gamma bands (30–48 Hz). Time-series were downsampled 4 times, and for each subject, five artifact free epochs of 4096 samples (13.1072 s) were selected (EvD) and further analyzed using Brainwave v0.9.58 [authored by C.S.; available at http://home.kpn.nl/stam7883/brainwave.html].

### 2.7 Functional connectivity

The phase lag index (PLI) was used to assess functional connectivity between the reconstructed signals in source-space. The PLI is a measure that is relatively insensitive to the effects of volume conduction (Stam et al., 2007). The synchronization between time-series is based on the consistency of the nonzero phase lag with respect to another signal. The instantaneous phase difference for each time sample is computed using the analytical signal concept and the Hilbert transform. The PLI characterizes the asymmetry in the distribution of instantaneous phase differences between two signals. The reason for using the asymmetry of this distribution as a measure of functional interactions is that a nonzero phase lag between these signals cannot be explained by volume conduction. The PLI ranges between 0 (no phase locking) and 1 (total synchronization). An index of the asymmetry of the phase distribution can be obtained from a time-series of phase differences Δφ(t_k_), k = 1 … N_s_ in the following way:

$$\text{PLI}=\left|\langle \text{sign}\left[\sin\left(\text{Δφ},\left({\text{t}}_{\text{k}}\right)\right)\right]\rangle \right|\text{,}$$

where the phase difference is defined in the interval [− π,π], <> denotes the mean value, and N_s_ is the number of samples. For each subject, the PLI was calculated for all possible ROI pairs. The functional connectivity within one of the predefined RSNs described below was then computed by averaging all PLI values between the ROIs within that sub-network.

### 2.8 Resting-state networks

Most RSNs described in literature are based on independent component analysis (ICA) applied to resting-state fMRI data. These ICA analyses suggest that the regions within each RSN are functionally closely coupled, at least at the level of BOLD-fMRI. Based on the assumption that such coupling should have a correlate in the MEG data (Brookes et al., 2011; Buxton et al., 1998; de Pasquale et al., 2012; Hipp et al., 2012; Nir et al., 2007), we defined MEG RSNs by selecting only those connections between the ROIs within literature-based RSNs. The review by Rosazza and Minati was used to define the RSNs, as it presents a complete overview of the RSNs with detailed information about their anatomical definitions (Rosazza and Minati, 2011). We selected four RSNs that are of interest for cognitive functioning in the domains that are frequently affected in LGG patients (Klein et al., 2012), namely the default mode network (DMN), the left and the right frontoparietal network (FPN), and the executive control network (see Table S1 for included ROIs). An overview of all RSNs and their definitions in terms of ROIs is provided in the supplementary material, as well as an analysis for a different definition of the RSNs (Table S1).

Most RSNs described in literature are based on independent component analysis (ICA) applied to resting-state fMRI data. These ICA analyses suggest that the regions within each RSN are functionally closely coupled, at least at the level of BOLD-fMRI. Based on the assumption that such coupling should have a correlate in the MEG data (Brookes et al., 2011; Buxton et al., 1998; de Pasquale et al., 2012; Hipp et al., 2012; Nir et al., 2007), we defined MEG RSNs by selecting only those connections between the ROIs within literature-based RSNs. The review by Rosazza and Minati was used to define the RSNs, as it presents a complete overview of the RSNs with detailed information about their anatomical definitions (Rosazza and Minati, 2011). We selected four RSNs that are of interest for cognitive functioning in the domains that are frequently affected in LGG patients (Klein et al., 2012), namely the default mode network (DMN), the left and the right frontoparietal network (FPN), and the executive control network (see Table S1 for included ROIs). An overview of all RSNs and their definitions in terms of ROIs is provided in the supplementary material, as well as an analysis for a different definition of the RSNs (Table S1).

### 2.9 Statistics

Statistics were computed using IBM SPSS statistics 18.0. The PLI is typically non-parametrically distributed between subjects, and the analyzed patient group contained a relatively small number of subjects. Wilcoxon signed ranks tests were therefore used to test whether PLI levels differed between T1 and T2 for mean PLI over all connections, and for the mean PLI within each RSN. Results were considered significant for p < 0.05, corrected for multiple testing using the false discovery rate (FDR) per frequency band. Possible differences between T1 and T2 regarding cognitive performance in any of the six domains were determined using Wilcoxon signed ranks tests because of the small sample size. When a significant RSN PLI alteration was found, we assessed possible correlations with cognitive performance in each of the six domains as a post-hoc analysis using Kendall's tau tests.

## 3 Results

**Fig. 1:**
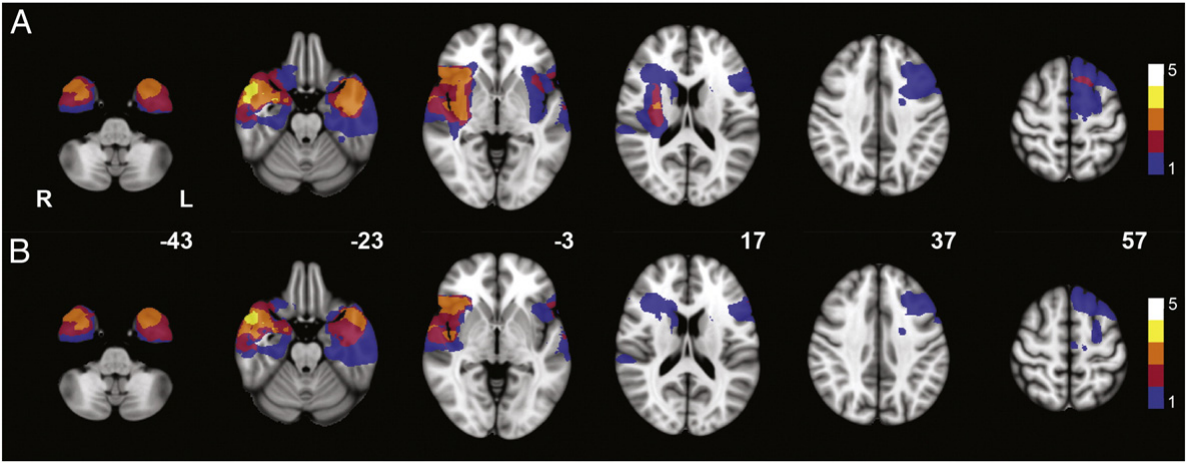
Localization of gliomas and resection cavities. Heatmap of lesion load for (A) glioma localization and (B) resection cavity localization of the study population on MNI standard brain template (MNI z-coordinates are given for each cross section). The legend shows the number of gliomas and resection cavities, respectively, that are represented by the colors in the figure. Note that most lesions are remote from the default mode network and right frontoparietal network shown in Figs. 2 and 3.

Ten patients with a WHO grade II glioma were included (Table 2; Fig. 1). Mean time between resection and T2 was 16 weeks (range 11–25 weeks). Neuropsychological data at both T1 (pre-resection) and T2 (post-resection) were available in eight patients. Wilcoxon signed ranks tests showed no differences in cognitive performance between T1 and T2 on a group level in any of the six cognitive domains (Table 3; table S2).

Ten patients with a WHO grade II glioma were included (Table 2; Fig. 1). Mean time between resection and T2 was 16 weeks (range 11–25 weeks). Neuropsychological data at both T1 (pre-resection) and T2 (post-resection) were available in eight patients. Wilcoxon signed ranks tests showed no differences in cognitive performance between T1 and T2 on a group level in any of the six cognitive domains (Table 3; table S2).

### 3.1 Functional connectivity analysis

**Fig. 2:**
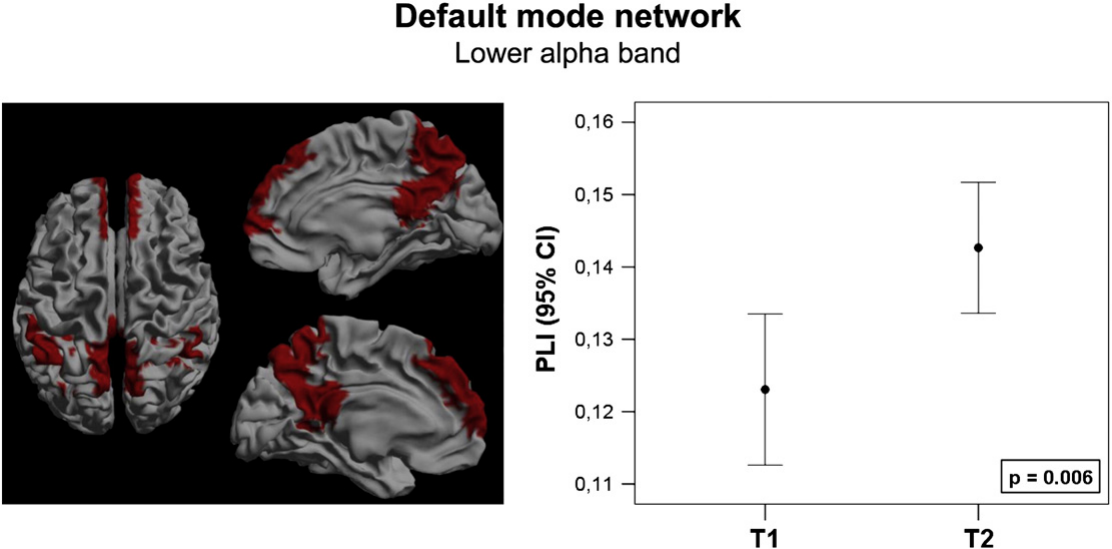
The default mode network, marked red in the left frame, showed increased connectivity in the lower alpha band after resection. Connectivity increase is presented as a 95% confidence interval at both time points. The p-value marks the significance of a Wilcoxon signed ranks test.

**Fig. 3:**
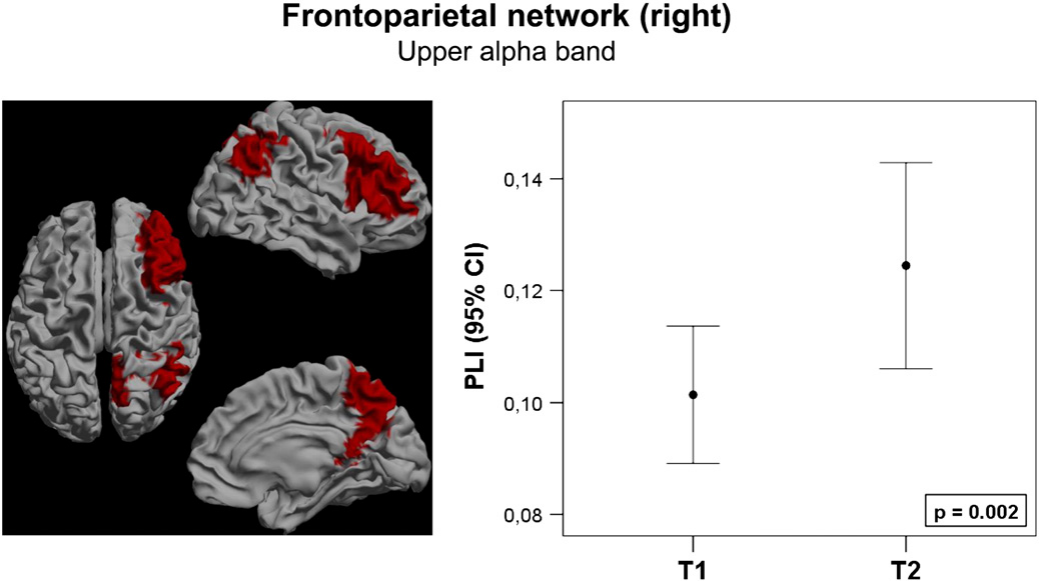
The frontoparietal network of the right hemisphere, marked red on the left frame, showed increased connectivity in the upper alpha band after resection. Connectivity increase is presented as a 95% confidence interval at both time points. The p-value marks the significance of a Wilcoxon signed ranks test.

First, we studied whether changes in global connectivity or connectivity of specific subnetworks after resective surgery can be detected using MEG. Therefore, global alterations in average PLI were determined using Wilcoxon paired signed ranks tests. No significant differences were found in any of the six frequency bands between T1 and T2. Furthermore, we determined PLI alterations for each frequency band in the four RSNs as defined by (Rosazza and Minati, 2011). After resection, patients showed higher PLI in the default mode network in the lower alpha band (z = − 2.599; p = 0.006; Fig. 2). Patients also showed higher PLI in the right FPN after resection in the upper alpha band (z = − 2.803; p = 0.003; Fig. 3). In this right FPN, a higher PLI was also found in the theta (z = − 2.191; p = 0.027) and lower alpha (z = − 2.191; p = 0.027) bands after resection, but only alteration in the upper alpha band remained significant after FDR correction. No significant alterations were found in the left FPN or in the ECN. This demonstrates that changes in connectivity can be detected using MEG.

**Fig. 4:**
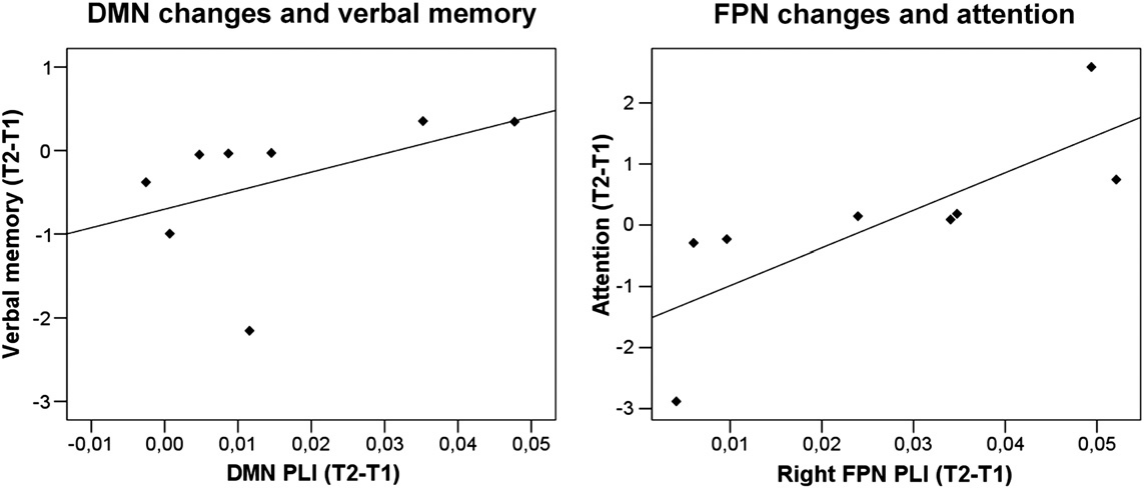
Correlations between lower alpha band DMN changes and verbal memory (left; tau = 0.571; R^2^ = 0.215; p = 0.048), and upper alpha band FPN changes and attention (right; tau = 0.857; R^2^ = 0.602; p = 0.003). Most patients show only small increases in RSN connectivity and small improvement in cognitive performance. Especially patients with no increase of RSN connectivity show deterioration of cognitive performance scores.

Second, to determine the relation between the MEG alterations in connectivity of specific subnetworks and changes in the corresponding cognitive domains, we assessed correlations (Kendall's tau) between cognitive performance and the RSNs that showed significant MEG connectivity changes after resection. In the lower alpha band, we found a positive correlation between DMN connectivity and verbal memory scores (tau = 0.571; p = 0.048; Fig. 4A). Similarly, upper alpha band right FPN connectivity correlated positively with attention (tau = 0.857; p = 0.003; Fig. 4B), working memory (tau = 0.571; p = 0.048) and executive functioning (tau = 0.643; p = 0.026). Of interest, patients with a relatively large cognitive improvement demonstrated a large increase of RSN connectivity (Fig. 4).

## 4 Discussion

The organization of the human brain can be described as a globally integrated network, in which local segregation is found in several subnetworks that are involved in different cognitive tasks (Bullmore and Sporns, 2009; Cole and Schneider, 2007; de Pasquale et al., 2012; Rosazza and Minati, 2011). In this study, surgical resection of LGG increased the connectivity of two of these networks, namely the default mode network (DMN) and the right frontoparietal network (FPN). Importantly, a large increase of resting-state network (RSN) activity was related to better post-surgical cognitive performance regarding attention, memory functioning, and executive functioning. This provides proof of principle that cognitive changes after resective surgery correspond with connectivity alterations in RSNs.

### 4.1 Resting-state networks and cognitive functioning

Previous cross sectional studies in healthy controls and stroke patients have elucidated the cognitive processes in which the RSNs that we analyzed are involved. Indeed, activity in the FPNs for memory and attention functioning was established in healthy controls (Corbetta et al., 2008; Dosenbach et al., 2007; Fornito et al., 2012). Two previous studies describe the effects of ischemic stroke on the FPN. Nomura and colleagues showed that the amount of damage to the FPN correlated with lower functional connectivity in that network, while connectivity in a control network was preserved (Nomura et al., 2010). This indicates that lesions in the FPN cause functional disturbances within this particular network, also to regions that were not directly damaged due to the lesion. Dubovik and colleagues showed in an EEG study that higher alpha band connectivity of the fronto-opercular cortex in stroke patients was related to verbal fluency and verbal working memory performance, while connectivity of the right inferior parietal cortex was related to spatial memory performance (Dubovik et al., 2012). Both these regions are part of the FPN. In the present longitudinal study, we found that increased alpha band right FPN connectivity was related to better performance in the attention, working memory, and executive function domains.

Our finding of better verbal memory in patients with increased DMN connectivity is somewhat different from previous findings. The DMN has mainly been described in fMRI studies as a network that is deactivated during task performance, while the resting-state level of connectivity is correlated to attention and working memory performance (see Broyd for an extensive review (Broyd et al., 2009)); decreased DMN connectivity was found in several diseases such as ADHD, autism and Alzheimer's disease. In contrast, increased connectivity in schizophrenia patients was related to excessive alertness (Broyd et al., 2009). Although increased connectivity within the DMN could thus be seen as a normalization after resection of the glioma, the correlation with verbal memory is not yet clear. We postulate that alterations in the DMN can have global effects on cognitive functioning because of its central role in the network, and we expect the correlation found between DMN connectivity and verbal memory to reflect this global effect.

### 4.2 Connectivity changes and resective surgery

To the best of our knowledge, this is the first study to show alterations in RSN connectivity after resective brain surgery. One previous study on the effects of tumor resection described that functional connectivity patterns after resection change in a complex way, demonstrating a decrease in interhemispheric theta band connectivity (Douw et al., 2008). Other work focused on the predictive value for surgical outcome of presurgical connectivity of the tumor region. When the resected area has a high alpha band functional connectivity, this suggests eloquence for language or motor functioning (Guggisberg et al., 2008; Martino et al., 2011; Tarapore et al., 2012). On the other hand, a central role or high connectivity in the presurgical network can also predict seizure freedom after resection, depending on the frequency of the analyzed oscillations (Ortega et al., 2008; Wilke et al., 2010). In our study, patients that showed the highest increase of alpha band RSN activity after surgery were also the ones with improved neuropsychological test scores.

In this study, patients demonstrated varying increase in connectivity of the DMN and the right FPN after a local surgical intervention. An important question is which factors determine why some patients show larger connectivity increases than others after resection, or, in a broader perspective, what determines the impact of a lesion or resection on the global brain network. One tempting hypothesis is that the level of increase in connectivity after resective surgery depends on the level that structures involved in the DMN and FPN are (directly or indirectly) affected by tumor infiltration or tumor mass effect. Many infiltrative gliomas in these patients involved long projection fibers, which are candidate structures subserving these networks. Alternatively, distant disinhibitive effects on the functional status of these networks cannot be ruled out until the structures substantiating these networks have been identified. Previous work based on MEG, EEG and fMRI has revealed global alterations in connectivity in the presence of a lesion, including the contralateral hemisphere (Bartolomei et al., 2006; Douw et al., 2009b, 2010; Gratton et al., 2012). Moreover, the impact of lesions in an anatomically realistic model of the human brain relied to a large extent on the importance, or ‘hub’ status, of the lesioned area in the anatomical network (Alstott et al., 2009). These studies therefore suggest that the preoperative connectivity of the region that is to be resected contains information on the postoperative functional changes that can be expected. In support of this hypothesis is previous work that has demonstrated that preoperative local connectivity is predictive for postoperative neurological functioning (Guggisberg et al., 2008; Martino et al., 2011; Tarapore et al., 2012). Future work should focus on the connectivity between the tumor region and RSNs to predict postoperative functional changes to guide surgical management decision.

### 4.3 Methodological considerations

The study of MEG functional connectivity and RSNs in source-space is a relatively new methodological approach. Combined EEG/fMRI registrations in healthy controls have indicated that particularly synchronized activity in the alpha band has a spatial correlate with RSNs such as the DMN, which was also the frequency range where our results were found (Jann et al., 2009). Previous MEG studies have validated these networks using Independent Component Analysis (ICA) applied to the Hilbert envelope of the MEG time-series (Brookes et al., 2011; de Pasquale et al., 2012; Hipp et al., 2012). We used the PLI to measure interactions between these validated predefined ROIs. Previous MEG studies have shown that the PLI is a valid measure of synchronization that is useful to characterize functional interactions related to cognitive processing in both healthy controls and glioma patients (Bosma et al., 2009; Douw et al., 2011). An important advantage of source-space MEG functional connectivity analysis is that it measures neuronal activity directly with a high temporal resolution, which is done in a standardized anatomical space using the beamformer approach, while functional MRI studies estimate neural activity based on an indirect measure of metabolic changes.

This study has some limitations. As part of our analysis approach we normalized the patients' MRIs to a template brain (and then labeled voxels using a standard (AAL) atlas), while our patients had altered anatomy due to the tumor, which may have influenced results. Moreover, for the postoperative beamformer analysis, data were projected to voxels at locations where no actual cortical tissue was present anymore due to the resection. However, we expect this to have minimal effects, as we used the voxel with the highest power as a representative source for each ROI, and voxels at locations without cortical tissue are not likely to show any activity apart from ‘leakage’ from surrounding sources (e.g. (Barnes et al., 2004)). Although a small number of patients were used, a correlation between the connectivity changes and cognitive changes was nevertheless detected. A larger cohort will be subject of future studies to further assess this relation.

In conclusion, improvement in cognitive performance was related to increased functional connectivity in resting-state networks after resective surgery of LGG, as detected by source-space MEG analysis. An increase in DMN connectivity in the lower alpha band was related with an increase in verbal memory performance, and an increase in FPN connectivity in the upper alpha band was related with an increase in attention, executive functioning, and working memory performance. This provides proof of principle that MEG functional connectivity is linked with cognitive outcome of resective surgery. These findings may be utilized in future studies to elucidate whether presurgical MEG resting-state connectivity can predict cognitive outcome, which would aid patient counseling and surgical management decisions.

The following are the supplementary data related to this article.

Supplementary material

Table S1 Resting-state networks.

Table S2 Differences in cognitive performance scores between T1 and T2.

Supplementary data to this article can be found online at http://dx.doi.org/10.1016/j.nicl.2012.10.007.

## Figures and Tables

**Table 1:** Cognitive test battery and corresponding domains.

Neuropsychological test	Corresponding cognitive domain
Concept shifting test	Executive functioning, psychomotor speed
Categoric word fluency task	Executive functioning
Rey auditory verbal learning test	Verbal memory
Stroop color-word test	Attention
Memory comparison test	Working memory
Letter-digit substitution test	Information processing, psychomotor speed

Tests were assessed as described by (Lezak, 1995), except for the Concept shifting test (Houx and Jolles, 1994).

**Table 2:** Patient characteristics.

Patient	Age	Gender	Lat.	Localization	Preop. vol.	Postop. vol.	EOR
1	20	F	R	Temp	7	0	99%
2	48	F	R	Front-Temp-Ins	72	8	89%
3	29	M	L	Front-Temp	68	4	94%
4	53	F	L	Temp	106	5	95%
5^a^	29	M	R	Front-Temp	78	25	68%
6	52	M	L	Temp	49	5	90%
7^a^	46	M	R	Front-Temp	80	19	76%
8	18	M	L	Front-Temp	37	1	98%
9	28	F	L	Front-Temp-Ins	56	10	82%
10	30	M	L	Front	30	11	63%

Patient characteristics. Lateralization and localization of the tumor are given, as well as tumor volumes (as determined with iPlan software, BrainLAB, Feldkirchen, Germany) and the extent of the resection. Abbreviations: Preop. vol = preoperative tumor volume (mL); Postop. vol. = preoperative tumor volume (mL); EOR = extent of resection; F = female; M = male; lat = lateralization of tumor; temp = temporal; front = frontal; ins = insula.

a No neuropsychological evaluation data available.

**Table 3:** Changes in cognitive performance.

Patient	Overall	Executive functioning	Verbal memory	Working memory	Information processing	Attention	Psychomotor speed
1	0.45	− 0.28	− 0.05	0.20	0.47	2.59	0.45
2	− 0.06	− 0.39	− 0.38	0.14	− 0.23	0.18	− 0.59
3	− 0.09	− 0.28	− 0.03	− 0.36	− 0.17	0.14	0.56
4	− 0.79	− 0.40	− 2.15	− 0.17	− 0.01	− 0.23	0.16
5	NA	NA	NA	NA	NA	NA	NA
6	− 0.02	− 0.51	0.35	0.06	0.57	− 0.29	− 0.86
7	NA	NA	NA	NA	NA	NA	NA
8	− 1.33	− 1.65	− 1.00	− 1.02	− 1.07	− 2.88	− 0.15
9	− 0.09	0.29	− 0.04	− 0.09	0.08	0.09	− 0.11
10	0.34	− 0.14	0.35	0.12	0.43	0.75	0.55

Individual cognitive performance scores are presented per domain. Values represent the difference of the z-scores between T1 and T2 (positive values indicate better performance after resection). NA = not available.
