# Supplementary material for: Connectivity in MEG resting-state networks increases after resective surgery for low-grade glioma and correlates with improved cognitive performance
Source: Neuroimage Clin. 2012 Nov 2;2:1–7. doi: 10.1016/j.nicl.2012.10.007 (PMC3777771; doi:10.1016/j.nicl.2012.10.007)
Supplement: Supplementary file 1 — Supplementary material [file mmc1.doc]

**Supplementary material**

Effects of RSN definitions

*Methods*

The RSNs as defined by Rosazza and Minati contain several shared connections (and ROIs) [1]. However, one could argue that each RSN should contain unique functional connections, because otherwise the interaction that is represented by a connection that is shared between RSNs is not specific for a certain cognitive domain. We therefore, in a separate analysis, also used a modified definition for the RSNs, one that allowed for a maximum of one ROI overlap between RSNs, such that each RSN contains a unique set of connections. An overview of all RSNs and their definitions in terms of ROIs is provided in table S1.

*Results*

We tested if the observed connectivity differences between T1 and T2 were also found using the alternative definition of RSNs as suggested by Tewarie and colleagues (Tewarie, in preparation; see table S1). Now, the lower alpha band showed a non-significant trend towards an increase after surgery (z = -1.836; p = 0.074) for the DMN. The right FPN still showed a significant increase after surgery in the upper alpha band (z = -2.191; p = 0.014), but no longer in the theta (z = -0.866; p = 0.432) and lower alpha (z = -0.663; p = 0.557) bands.

*Discussion*

We used predefined RSNs in this study, consisting of ROIs that are consistently described in literature [1-3]. As a sensitivity analysis, a second analysis was performed with an adjusted definition of RSNs proposed by Tewarie and others, that permits only one ROI overlap between RSNs, hereby preventing connections to be part of multiple RSNs. In this analysis, theta band and lower alpha band connectivity increases in the FPN were no longer significant. Similarly, the change in DMN PLI was no longer significant after adding of the anterior cingulate ROI to the DMN. We suggest that the current study should be seen as a framework for this type of analysis rather than an absolute definition of the RSNs, which should be based on a large cohort of healthy controls. However, our findings do indicate that the overlapping connections between RSNs may be of functional importance for different cognitive processes, and that a strict separation of the RSNs should only be applied when empirically justified. The fact that cognitive tasks are always performed simultaneously with other tasks indicates that these shared connections may yield crucial information for the integration of multiple processes [4].

**References**

1. Rosazza, C. and L. Minati, *Resting-state brain networks: literature review and clinical applications.* Neurol Sci, 2011. **32**(5): p. 773-85.

2. Brookes, M.J., et al., *Investigating the electrophysiological basis of resting state networks using magnetoencephalography.* Proc Natl Acad Sci U S A, 2011. **108**(40): p. 16783-8.

3. Habeck, C., et al., *Can the default-mode network be described with one spatial-covariance network?* Brain Res, 2012. **1468**: p. 38-51.

4. van den Heuvel, M.P., et al., *High-cost, high-capacity backbone for global brain communication.* Proc Natl Acad Sci U S A, 2012. **109**(28): p. 11372-7.
