# Supplementary material for: Connectivity in MEG resting-state networks increases after resective surgery for low-grade glioma and correlates with improved cognitive performance
Source: Neuroimage Clin. 2012 Nov 2;2:1–7. doi: 10.1016/j.nicl.2012.10.007 (PMC3777771; doi:10.1016/j.nicl.2012.10.007)
Supplement: Table S1 — Resting-state networks. [file mmc2.doc]

**Table S1** Resting-state networks

| **Resting-state network** | **Corresponding AAL atlas ROIs**  **(Rosazza and Minati, 2011)** | **Corresponding AAL atlas ROIs**  **(1 ROI overlap)** |
| --- | --- | --- |
| Default mode network | Precuneus, posterior cingulate gyrus, inferior parietal gyrus, medial prefrontal gyrus | Precuneus, posterior cingulate gyrus, anterior cingulate gyrus*, inferior parietal gyrus, medial prefrontal gyrus |
|
| Executive control | Medial frontal cortex, superior frontal gyrus, anterior cingulate gyrus | Medial frontal cortex, superior frontal gyrus, anterior cingulate gyrus |
| Frontoparietal (left/right) | Inferior frontal gyrus pars triangularis, inferior frontal gyrus pars opercularis*, medial frontal gyrus, precuneus*, inferior parietal gyrus, angular gyrus | inferior frontal gyrus pars triangularis, medial frontal gyrus, inferior parietal gyrus, superior parietal gyrus*, angular gyrus |
|

Definitions of the analyzed RSNs. Data that were presented as main results in the paper were based on the ROI definition of Rosazza and Minati. However, a slight modification to this definition was proposed by Tewarie and others (in preparation), which prevents overlap of connections between RSNs (right column). Our data were also analyzed using this definition. Differences between both definitions were marked with *.
