# Supplementary material for: Connectivity in MEG resting-state networks increases after resective surgery for low-grade glioma and correlates with improved cognitive performance
Source: Neuroimage Clin. 2012 Nov 2;2:1–7. doi: 10.1016/j.nicl.2012.10.007 (PMC3777771; doi:10.1016/j.nicl.2012.10.007)
Supplement: Table S2 — Differences in cognitive performance scores between T1 and T2. [file mmc3.doc]

**Table S2** Differences in cognitive performance scores between T1 and T2

| **Cognitive domain** | **Mean** | **SD** | **p-value** |
| --- | --- | --- | --- |
| Executive functioning (T2-T1) | 0.42016 | 0.55437 | 0.055 |
| Verbal memory (T2-T1) | 0.36802 | 0.83971 | 0.250 |
| Working memory (T2-T1) | 0.13986 | 0.40295 | 0.641 |
| Information processing (T2-T1) | -0.00823 | 0.52933 | 0.844 |
| Attention (T2-T1) | -0.04317 | 1.49959 | 0.945 |
| Psychomotor speed (T2-T1) | -0.00094 | 0.53018 | 1.000 |

Wilcoxon signed ranks tests comparing patients’ cognitive performance z-scores before and after tumor resection. No significant differences were found.
